# Competing bonding and anharmonicity control piezoelectricity and thermal transport in janus BrSbX monolayers

**DOI:** 10.1039/d5ra08461j

**Published:** 2026-01-30

**Authors:** Viet-Ha Chu, Quang Hai Nguyen, Mai An Pham, Minh Tuan Luong, Truong-Tho Pham, Duc-Long Nguyen

**Affiliations:** a Department of Physics, TNU-University of Education Thai Nguyen 250000 Vietnam chuvietha@tnue.edu.vn; b HaNoi University of Civil Engineering Vietnam; c Laboratory of Magnetism and Magnetic Materials, Science and Technology Advanced Institute, Van Lang University Ho Chi Minh City Vietnam; d Simulation in Materials Science Research Group, Science and Technology Advanced Institute, Van Lang University Ho Chi Minh City Vietnam nguyenduclong@vlu.edu.vn; e Faculty of Applied Technology, Van Lang School of Technology, Van Lang University Ho Chi Minh City Vietnam

## Abstract

We show how competing bonding strength and lattice anharmonicity govern piezoelectricity and thermal transport in Janus 1T-BrSbX monolayers (X = S, Se, Te). Using density-functional theory and density-functional perturbation theory, combined with machine-learning-accelerated third-order force constants and Boltzmann transport calculations, we map composition property relationships within a single symmetry family. A bonding analysis (COHP) reveals a monotonic reduction in Sb–X covalency from S → Te, which tracks the piezoelectric stress response *e*_11_ and yields in-plane strain coefficients *d*_11_ = 41.0, 22.1, 6.3 pm V^−1^ for BrSbS, BrSbSe, and BrSbTe, respectively. In thermal transport, longitudinal-acoustic group velocities decrease from ∼3.41 to <2.78 km s^−1^ (S → Te), but mode-averaged Grüneisen parameters diminish more strongly, so phonon lifetimes dominate the trend: *κ*_L_(300 K) = 5.94, 9.39, 12.32 W m^−1^ K^−1^ for S, Se, and Te, respectively. All monolayers are mechanically and dynamically stable. Together, these results establish a practical design rule: strengthening directional Sb–X covalency enhances *d*_11_, while reduced anharmonicity raises *κ*_L_; the chalcogen thus provides a clean chemical knob to balance electromechanical coupling against heat transport in 2D Janus pnictogen chalcogenides.

## Abstract 2

We show how competing bonding strength and lattice anharmonicity govern piezoelectricity and thermal transport in Janus 1T-BrSbX monolayers (X = S, Se, Te).

## Graphical abstract

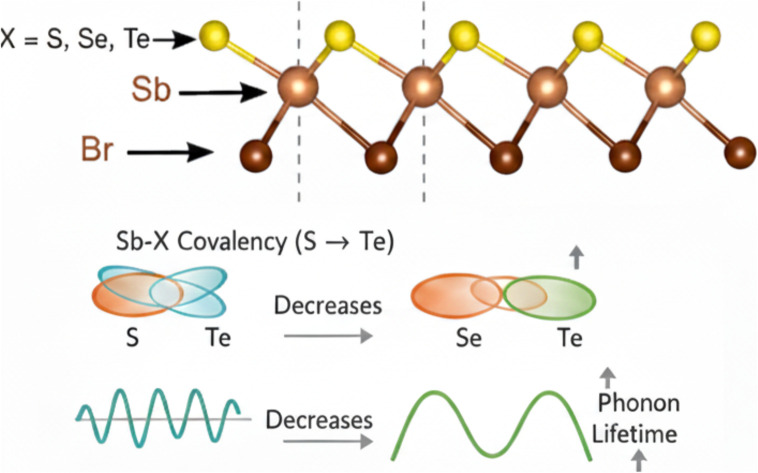

## 1 Introduction

Two-dimensional crystals have emerged as a fertile platform for the discovery of properties that are absent in bulk systems, owing to their strong quantum confinement, broken symmetry, and tunable electronic structure.^1,2^ Among these, Janus monolayers are formed when the two out-of-plane surfaces contain different atomic species, which breaks mirror symmetry and induces intrinsic dipole moments. This structural polarity modifies band alignment, vibrational modes, and dielectric responses, making Janus systems attractive for nanoscale energy harvesting, sensing, and electronic devices.^3–12^ Studies across diverse chemical families, including transition metal dichalcogenides, pnictogen chalcogenides, and nitrides, have confirmed that compositional asymmetry is a robust route to multifunctionality.

Piezoelectricity is one of the most direct consequences of broken inversion symmetry in two-dimensional materials. Calculations on single-layer transition metal dichalcogenides and oxides reported sizable piezoelectric coefficients once symmetry was removed.^13–15^ Later works demonstrated very large out of plane responses in Janus tellurene compounds,^16^ and ultrahigh out of plane piezoelectric coefficients of several pm V^−1^ in Janus group IV trichalcogenides, accompanied by a giant Rashba effect.^17^ Dimple and co-workers showed that group IVB Janus dichalcogenides possess piezoelectric coefficients exceeding 20 pm V^−1^ along with robust electron mobility.^18^ Nandi *et al.* predicted gigantic shear piezoelectricity in group IVA Janus dichalcogenides, with *d*_15_ values greater than 700 pm V^−1^ under uniaxial tensile strain.^19^ Zhang *et al.* reported Janus ZnXY_2_ monolayers exhibiting both high flexibility and significant piezoelectric coefficients up to 18 pm V^−1^.^20^ Guo *et al.* demonstrated that small biaxial strain can further enhance the coefficients in AsP Janus monolayers.^21^ Oxygen functionalization in Janus MXenes has been shown to yield out of plane coefficients above 25 pm V^−1^.^22^ Binary Janus systems and Li-based compounds extend these possibilities, with coefficients surpassing 30 pm V^−1^ in some cases^23^?. MoXSiN_2_ nitrides have also been identified with combined in plane and out of plane piezoelectricity exceeding that of conventional dichalcogenides.^24^ Collectively these studies confirm that structural asymmetry provides a consistent pathway to very large piezoelectric responses across chemistries.^25,26^

Janus materials have also been explored as thermoelectrics, where efficient performance requires high electronic transport combined with suppressed lattice thermal conductivity. Jakhar *et al.* studied Janus *β* PdXY monolayers and reported highly anisotropic figures of merit with *ZT* values approaching 3 at high temperature, driven by lattice thermal conductivities below 1 W m^−1^ K^−1^.^27^ Das *et al.* investigated ZrXY Janus monolayers and found *ZT* values above 2, supported by favorable band alignment and reduced *κ*.^28^ In bulk, Roychowdhury *et al.* demonstrated ultrahigh thermoelectric performance in AgSb_1−*x*_Zn_*x*_Te_2_ with *ZT* = 2.6 at 700 K along with improved mechanical stability.^29^ Recent studies of marcasite-type thermoelectrics revealed figures of merit above 2.4 due to converged bands and strong phonon scattering.^30^ These results indicate that chemical asymmetry and bonding anisotropy in Janus systems provide systematic control over phonon scattering and electronic transport, making them strong candidates for efficient thermoelectrics. It should be emphasized that reduced lattice thermal conductivity is not a universal consequence of Janus asymmetry. In many Janus monolayers, the lattice thermal conductivity *κ*_L_ falls between those of the two centrosymmetric parent limits because Janus functionalization simultaneously perturbs harmonic phonon dispersions (thus modifying phonon group velocities) and anharmonic phonon–phonon scattering (thus modifying phonon lifetimes). The resulting *κ*_L_ therefore reflects a competition between *v*_g_ and *τ*. In selected systems, enhanced anharmonicity outweigh harmonic effects and drive *κ*_L_ below both symmetric parents, as reported for Sn_2_PAs^31^ and PtSTe.^32^ Strong anharmonicity accompanied by low *κ*_L_ has also been identified in the Janus Rashba semiconductor RbKNaBi.^33^ This broader landscape motivates mechanism-based analysis in which anharmonicity is evaluated explicitly rather than inferred solely from phonon softening.

In Janus materials, anharmonic phonon–phonon interactions frequently regulate thermal transport in addition to harmonic phonon dispersions. Guo *et al.* showed that in Janus InXO monolayers, large mode-dependent Grüneisen parameters suppress phonon lifetimes, leading to ultralow lattice thermal conductivity below 1 W m^−1^ K^−1^ even with stiff dispersions.^34^ Su *et al.* studied Janus niobium based monolayers and identified strong anharmonic scattering as the dominant mechanism for reduced conductivity.^35^ Ma *et al.* predicted large out of plane piezoelectricity coupled with anharmonic suppression of thermal conductivity in Janus systems.^36^ These results indicate that the simple assumption that softer phonons imply lower conductivity is not generally valid, and that anharmonic effects must be systematically evaluated to understand thermal transport in Janus crystals. Apart from thermoelectricity and piezoelectricity, Janus monolayers exhibit a variety of multifunctional characteristics. Rawat *et al.* highlighted how nanoscale Janus interfaces modify electronic and ionic transport in energy materials.^37^ Guo *et al.* reported that intrinsic piezoelectricity can coexist with nontrivial band topology in Janus compounds.^38^ An *et al.* identified superconductivity and topological properties in related layered systems.^39^ Afsari *et al.* studied Janus membranes and demonstrated selective transport suitable for distillation processes.^40^ Beknalkar *et al.* critically evaluated the role of piezoelectric coupling in supercapacitors, outlining recent advances in hybrid energy storage.^26^ Yu *et al.* synthesized mesoporous Janus nanoparticles with tunable catalytic properties for chemical applications.^41^ Together these reports illustrate the diversity of functionalities that emerge from compositional polarity at the atomic scale.

A mechanistic understanding of how bonding covalency and anharmonic phonon scattering co-evolve within a chemically consistent Janus family, and how this co-variation simultaneously governs piezoelectric response and lattice thermal conductivity, is still lacking. Previous studies often optimized piezoelectric coefficients or minimized lattice thermal conductivity in isolation, leaving their co-variation largely unexplored.^1,2,5^ Motivated by these gaps, we investigate the 1T-BrSbX (X = S, Se, Te) Janus family as a controlled model system to elucidate how bonding covalency and anharmonicity jointly regulate piezoelectric and thermal transport properties. The corresponding symmetric parent structures adopt the centrosymmetric *P*3̄*m*1 phase and are therefore non-piezoelectric, whereas Janus functionalization breaks out-of-plane mirror symmetry and introduces an intrinsic polarity without altering the underlying 1T lattice topology. Importantly, the S–Se–Te substitution preserves the same structural framework and symmetry class, enabling a clean comparison in which trends can be attributed primarily to systematic tuning of Sb–X bonding character and mass contrast rather than to variations across unrelated material families. Experimentally, Janus monolayers such as MoSSe^42^ and WSSe^43^ have been realized *via* selective top-layer substitution, suggesting feasible synthesis pathways for BrSbX. Using first-principles calculations, we quantify how chalcogen substitution modulates elastic stiffness, phonon dispersions, and anharmonic phonon scattering, and we reveal how these factors co-determine the electromechanical response and lattice thermal conductivity in this family.

## 2 Computational methods

All first principles calculations were carried out using density functional theory (DFT)^44,45^ as implemented in the Quantum ESPRESSO package.^46–48^ The generalized gradient approximation of Perdew, Burke, and Ernzerhof (PBE) form was used to treat the exchange–correlation functional.^49^ A vacuum spacing of 15 Å along the *z* axis was introduced to remove spurious interlayer interactions due to periodic boundary conditions. Atomic positions and lattice constants were fully relaxed until the total energy converged within 10^−6^ Ry and the forces on each atom were less than 10^−3^ eV Å^−1^. The conjugate gradient algorithm was used for structural optimization, and the resulting Hellmann–Feynman forces ensured accurate equilibrium configurations. Kinetic energy cutoff for wavefunctions and charge density cutoffs were set to 60 Ry and 480 Ry, respectively. The Brillouin zone was sampled using a 13 × 13 × 1 Monkhorst–Pack grid, which provided well converged total energies and stress tensors. All calculations employed scalar-relativistic norm-conserving pseudopotentials without explicit spin–orbit coupling (SOC). SOC is expected to affect the detailed band dispersions and band gaps, but to have a much smaller impact on structural parameters, low-frequency phonons, and the elastic and piezoelectric tensors that are the primary focus of this work.

Phonon dispersions were calculated using density functional perturbation theory.^50^ Harmonic and third order interatomic force constants were computed with the Phonopy and Phono3py packages.^51,52^ The lattice thermal conductivity was obtained by solving the phonon Boltzmann transport equation within the single mode relaxation time approximation (RTA) on a 101 × 101 × 1 *q*-point mesh. Convergence of *κ*_L_ with respect to the *q*-point mesh was checked by comparing results at 300 K for 96 × 96 × 1, 101 × 101 × 1 and 114 × 114 × 1 grids. *κ*_*xx*_ does not change for all compounds, indicating that the 101 × 101 × 1 grid used throughout is sufficient. The phonon BTE was solved within the single-mode relaxation-time approximation (RTA); for the moderate *κ*_L_ values and strong anharmonic scattering found here, RTA is expected to provide *κ*_L_ values close to the full iterative solution (around 5%), typically slightly underestimating *κ*_L_ and therefore yielding conservative estimates. Mode resolved Grüneisen parameters were evaluated to quantify anharmonic phonon–phonon interactions.

To accelerate sampling of anharmonic interactions and enable larger supercells, machine learning interatomic potentials were employed. Moment tensor potentials (MTPs) were trained with the MLIP package^53^ using datasets from *ab initio* molecular dynamics trajectories. Training configurations were generated for BrSbX (X = S, Se, Te) monolayers using 5 × 5 × 1 supercells, a 3 × 3 × 1 *k*-point grid, and a 1 fs timestep. Two molecular dynamics runs were performed from 0–200 K and 200–700 K, each consisting of 1000 steps, yielding 1800 structures for training and 200 for testing. These potentials provided reliable forces for nearly 1900 structures, which were subsequently used to compute harmonic and anharmonic force constants. Corrections of the second order interatomic force constants were performed using the Hiphive package,^54^ which enforces rotational invariance and the Born–Huang sum rules, ensuring the quadratic character of the out of plane acoustic branch near the *Γ* point. This approach, combined with previous demonstrations of efficient ML based force constant extraction in 2D systems,^55^ guarantees robust evaluation of anharmonic scattering. In the present work, only three-phonon scattering processes are included in the solution of the phonon Boltzmann transport equation; four-phonon interactions are neglected. This approximation is expected to be reasonable for the BrSbX monolayers because their room-temperature lattice thermal conductivities are in the moderate range 5–12 W m^−1^ K^−1^, where three-phonon Umklapp scattering typically dominates heat transport. The reported *κ*_L_ values should therefore be interpreted as upper bounds that would be modestly reduced, particularly at elevated temperatures, upon inclusion of four-phonon scattering^56^

For 2D materials, the thermal conductivity values were normalized according to *L*_*z*_/*d*, where *L*_*z*_ is the supercell length along the *z* direction and *d* is the sum of the monolayer thickness and van der Waals radii of the terminating atoms. This procedure provides consistent comparison of intrinsic thermal transport between different BrSbX compositions.

### 2.1 Elastic and piezoelectric properties

The second order elastic constants (*C*_*ij*_) and piezoelectric tensors (*e*_*ijk*_) were obtained using density functional perturbation theory (DFPT) as implemented in Quantum ESPRESSO.^46–48,50^ Within Voigt notation, the stress tensor *τ*_*α*_ is related to the strain tensor *η*_*β*_ through Hooke's law,1
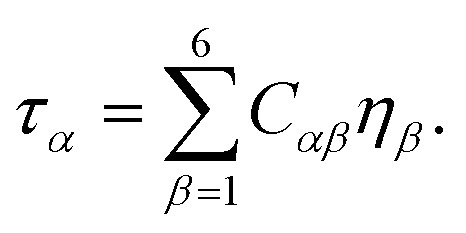
The elastic constants can equivalently be obtained from the curvature of the total energy with respect to strain,2
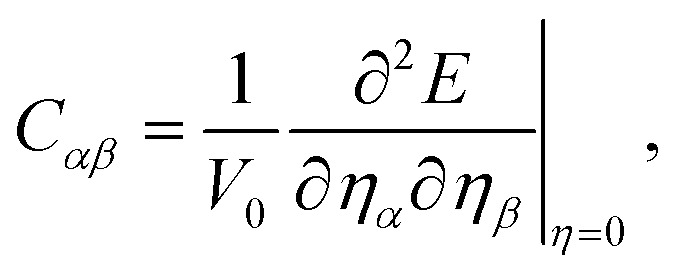
where *V*_0_ is the equilibrium volume.

The linear coupling between mechanical strain and polarization *P* is described by the piezoelectric effect. The stress tensor *e*_*ijk*_ and the strain tensor *d*_*ijk*_ are defined as3
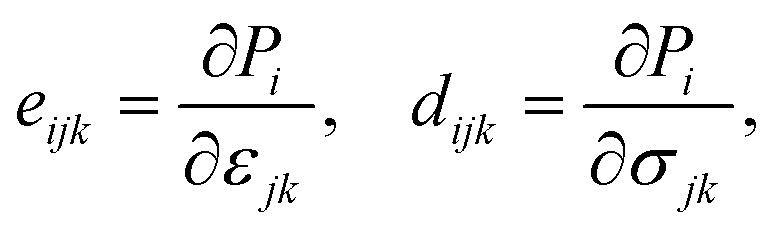
with *ε* and *σ* the strain and stress tensors, respectively. These are connected through the elastic compliance tensor *S* = *C*^−1^. For the trigonal symmetry (point group 3*m*) of BrSbX, the in-plane piezoelectric strain coefficient *d*_11_ is obtained from4
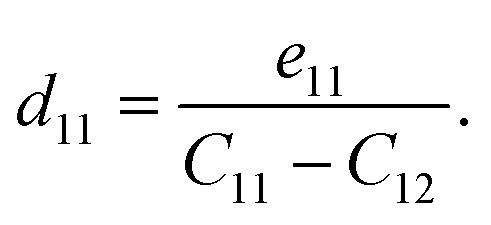


### 2.2 Machine learning interatomic potentials and thermal transport

Direct calculation of lattice thermal conductivity *κ*_L_ from DFT is computationally expensive because it requires third-order interatomic force constants (IFCs) describing three-phonon scattering.^50–52^ To address this, we employed a hybrid approach combining DFT with machine learning interatomic potentials. Specifically, moment tensor potentials (MTPs) were trained using the MLIP package,^53^ with training datasets constructed from *ab initio* molecular dynamics trajectories. This strategy has been demonstrated to accelerate force constant extraction for thermal transport in two-dimensional systems.^55^ To ensure physical invariance, second-order IFCs were corrected with the Hiphive package,^54^ enforcing rotational and translational symmetries.

The phonon Boltzmann transport equation was solved within the single-mode relaxation time approximation using Phono3py.^51,52^ The lattice thermal conductivity tensor was computed as5
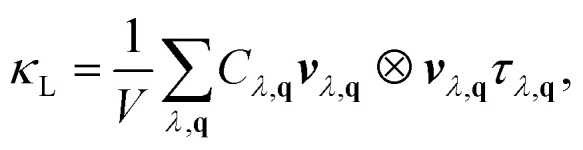
where *V* is the unit cell volume, *C*_*λ*,**q**_ the modal heat capacity, ***v***_*λ*,**q**_ the phonon group velocity, and *τ*_*λ*,**q**_ the phonon lifetime from third-order scattering. The group velocity was obtained from ***v***_*λ*,**q**_ = ∇_**q**_*ω*_*λ*,**q**_.

The degree of lattice anharmonicity was quantified through the Grüneisen parameter,6
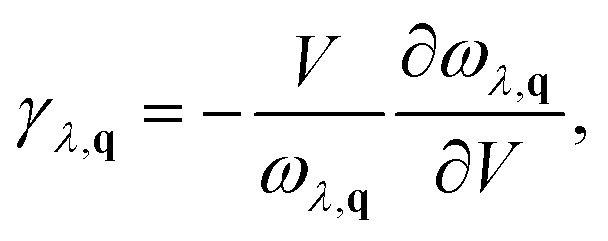
where large values of *γ* correspond to strong anharmonic coupling, shorter phonon lifetimes, and lower *κ*_L_.

## 3 Results and discussion

### 3.1 Structural stability and electronic characteristics

The BrSbX (X = S, Se, Te) monolayers investigated in this work adopt a centrosymmetry-broken trigonal crystal structure belonging to the *P*3*m*1 space group. Each compound forms a single two-dimensional layer stacked along the (0, 0, 1) direction, comprising edge-sharing SbX_3_Br_3_ octahedra. The antimony atom is coordinated by three equivalent chalcogen atoms (X) and three equivalent Br atoms in a slightly distorted octahedral environment. Although all three systems share the same symmetry and atomic topology, systematic variations in bond length and electronic environment emerge across the chalcogen series, intimately affecting their physical properties.

In BrSbS, the length of the Sb–S bond is 2.68 Å, indicating strong covalent bonding, while the Sb–Br bond measures 3.01 Å. This short Sb–S bond correlates with higher optical phonon frequencies, as will be discussed in the context of vibrational modes. Moving to BrSbSe, the larger chalcogen atom increases the SbSe bond to 2.81 Å, while the Sb–Br bond remains nearly unchanged at 3.01 Å, reflecting the relatively fixed position of Br as a terminal species. In BrSbTe, the SbTe bond expands further to 2.99 Å, and the Sb–Br bond increases slightly to 3.03 Å, consistent with the larger ionic radius and weaker bonding character of Te. In particular, while BrSbS and BrSbSe both preserve the *ω*-type trigonal motif, BrSbTe adopts a structure derived from the CdI_2_-type 1T polytype, although still maintaining the same *P*3*m*1 space group and planar topology.

**Fig. 1:**
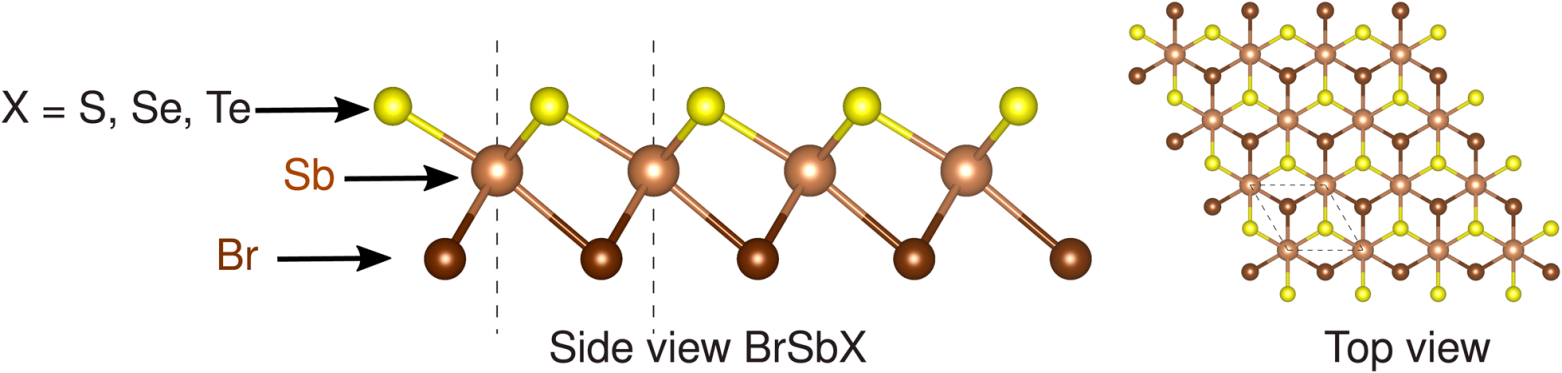
Side and top views of the Janus BrSbX (X = S, Se, Te) monolayer structure. In the side view, the atomic layers exhibit vertical asymmetry with halogen (Br) atoms at the bottom, antimony (Sb) atoms in the middle, and chalcogen atoms (X = S, Se, Te) on the top. Dashed lines indicating the unit cell boundaries.

The Janus 1T-BrSbX monolayers are not strictly planar: Br, Sb, and X reside at distinct out-of-plane coordinates (vertical asymmetry; Fig. 1) while preserving the in-plane 1T lattice symmetry, and the lack of inversion symmetry, arising from the vertical chemical asymmetry between the chalcogen and halogen planes, renders the systems intrinsically piezoelectric. This broken mirror symmetry is similar to that seen in Janus transition metal dichalcogenides, such as MoSSe or MoSTe, although realized here in a group V-halide chalcogenide framework. Importantly, the differing Sb–X and Sb–Br bond lengths and characters create anisotropy in both the local bonding environment and the lattice dynamics. This leads to different phonon dispersions and bonding stiffness, which ultimately manifests itself in sharp variations in both the piezoelectric coefficient in the plane *d*_11_ and the thermal conductivity of the lattice *κ*_L_, as detailed in subsequent sections. The stronger covalency and shorter bond lengths in BrSbS facilitate higher-frequency in-plane polarized phonon modes that are more effective in generating piezoelectric polarization under strain, accounting for its substantially higher *d*_11_ value compared to BrSbSe and BrSbTe. In contrast, the longer and weaker SbTe bonds in BrSbTe support lower group velocities but also result in weaker anharmonic scattering-leading to a comparatively higher thermal conductivity. Thus, the crystal structure provides not only the symmetry framework required for piezoelectricity but also the chemical foundation that dictates the extent and efficiency of electromechanical and thermal responses in this family. To assess thermodynamic stability, we evaluated the formation enthalpy of 1T-BrSbX monolayers relative to the constituent bulk elemental phases Sb, Br, and X (X = S, Se, Te). All three compositions exhibit negative formation energies −0.696, −0.728 and −0.677 per formula unit for 1T-BrSbS, 1T-BrSbSe, 1T-BrSbTe respectively, indicating stability against decomposition into the elements. In addition, the 1T polytype is more stable than the corresponding 2H stacking by −0.415,-0.415, and −0.422 eV/f.u. for BrSbS, BrSbSe, and BrSbTe, respectively. This energy hierarchy rationalizes our focus on the 1T Janus structure. Finite-temperature stability was examined implicitly through the *ab initio* molecular dynamics trajectories used to generate training data for the MLIP (Sec. 2.2). In 5 × 5 × 1 supercells evolved up to 700 K for 2× 10^3^ timesteps with a 1 fs timestep, the Janus BrSbX layers retain their crystallographic symmetry and bonding topology, with no evidence of reconstruction, desorption of the Br or chalcogen planes, or phase separation. Together with the absence of imaginary modes in the phonon spectra (Fig. 4), these results support both dynamical and thermal stability of the 1T-BrSbX monolayers under ambient and moderately elevated temperatures.

### 3.2 Chemical bonding analysis

**Fig. 2:**
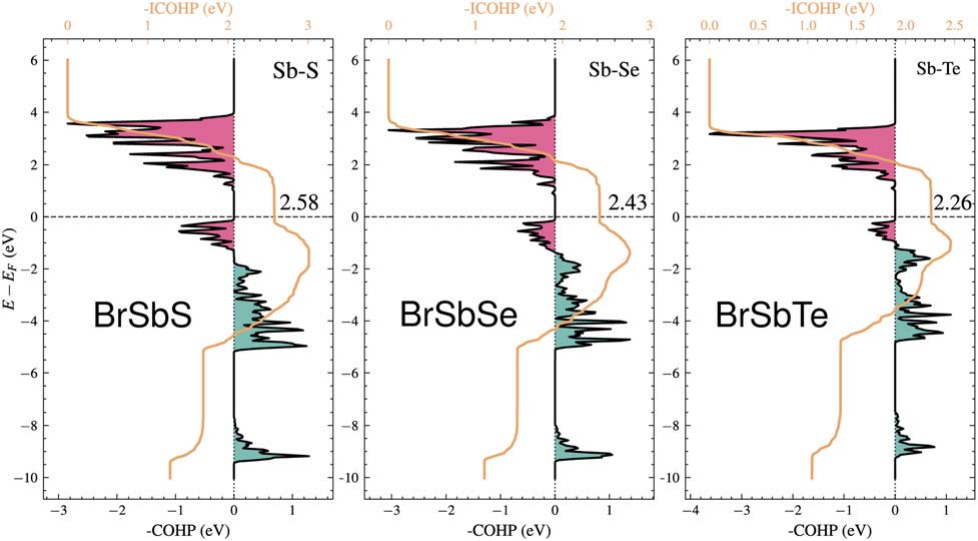
Crystal orbital Hamilton population (COHP) plots for the Sb–X (X = S, Se, Te) bonds in monolayer BrSbX compounds: (left) BrSbS, (middle) BrSbSe, and (right) BrSbTe. The bonding (negative COHP) and antibonding (positive COHP) contributions are shown with the black curves, and the corresponding integrated COHP values are overlaid in orange. The Fermi energy (*E*_F_) is set to 0 eV. Pink and teal shaded regions represent antibonding and bonding states, respectively. A progressive weakening of the Sb–X bond is observed from BrSbS to BrSbTe, with integrated COHP values decreasing from 2.58 eV to 2.26 eV, in line with the reduction in covalency and increasing bond length across the chalcogen series. This bonding evolution directly influences the piezoelectric response and vibrational properties discussed in the main text.

To clarify the variation in the piezoelectric response across the BrSbX series (X = S, Se, Te), a comparative Crystal Orbital Hamilton Population (COHP) examination was carried out for Sb–X and Sb–Br bonds. In what follows, the reported quantities are the magnitudes of the integrated COHP, |iCOHP| (eV/bond), so that larger values indicate stronger covalency. For the Sb–X bonds, |iCOHP| decreases monotonically from S to Te, with representative values of 2.58 for Sb–S in BrSbS, 2.43 for Sb–Se in BrSbSe, and 2.26 for Sb–Te in BrSbTe. By contrast, the Sb–Br interaction varies only weakly, with |iCOHP| changing from 1.22 (BrSbS) to 1.19 (BrSbSe) and 1.14 (BrSbTe), indicating that the halogen coordination remains comparatively stable across the series. This selective weakening of Sb–X covalency is mirrored by a systematic reduction of the in-plane piezoelectric strain coefficient *d*_11_, which follows the sequence 41.0 pm V^−1^ (BrSbS) → 22.1 pm V^−1^ (BrSbSe) → 6.3 pm V^−1^ (BrSbTe). Taken together, the comparative COHP trends and the piezoelectric coefficients support a picture in which the primary control parameter for *d*_11_ within this family is the strength of the Sb–X bond, whereas Sb–Br acts mainly as a structural termination with limited variation and no dominant role in the in-plane piezoelectric behavior. For completeness, COHP curves for Sb–Br are provided in Fig. S2 of the SI, and the overall evolution is consistent with a progressive weakening of directional covalency from S to Te that moderates the polarization response under in-plane strain (Fig. 2).

**Fig. 3:**
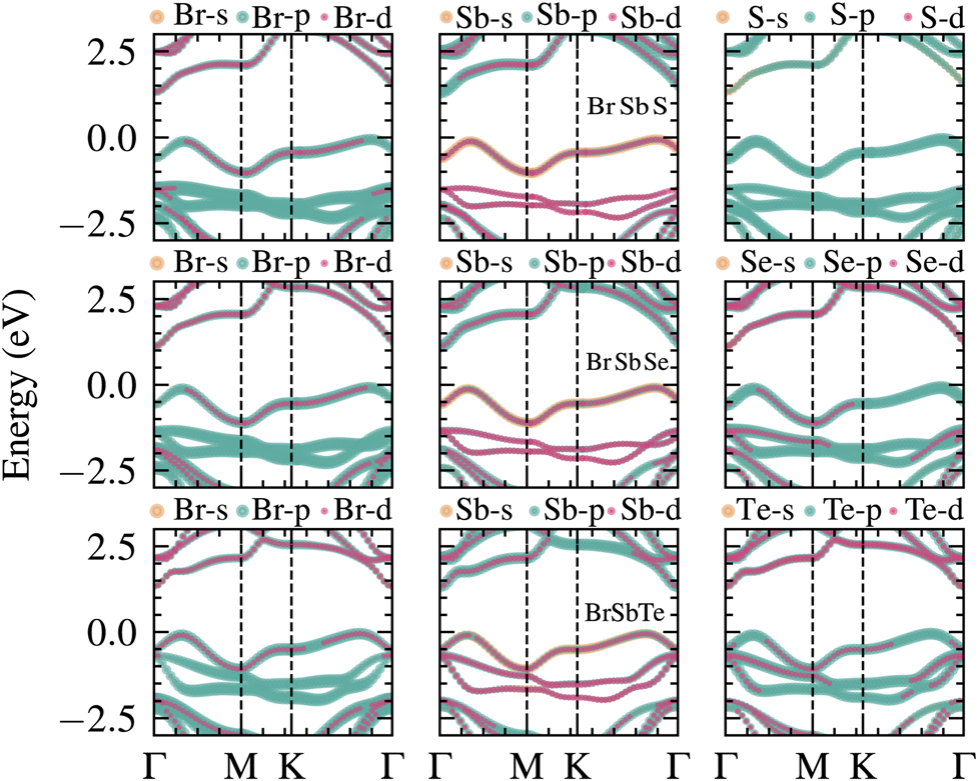
Orbital-projected electronic band structures of monolayer BrSbX (X = S, Se, Te) compounds. One compound is represented by each row: (top) BrSbS, (middle) BrSbSe, and (bottom) BrSbTe. The columns show the contributions from Br (left), Sb (center), and X = S, Se, Te (right) atoms, with s, p, and d orbital characters represented by distinct colors. The band structures are plotted along the high-symmetry path *Γ*–M–K–*Γ*, with the Fermi level set to 0 eV. Across the series, the valence band maximum (VBM) is dominated by Sb-p and X-p orbitals, while Br-p states contribute more weakly and lie deeper in energy. Notably, the increasing chalcogen atomic size (S → Se → Te) induces a downward shift of the conduction bands and modifies orbital hybridization, especially in the vicinity of the VBM and conduction band minimum (CBM), reflecting systematic tuning of electronic structure through chemical substitution.

The projected electronic band structures of BrSbX in Fig. 3 provide detailed information on the orbital nature of states near the Fermi level and their evolution in the BrSbX series. Along the high symmetry points of the BZ, the valence-band maximum carries dominant Sb-p character with substantial X-p admixture in all three compounds, while a finite Sb-s component is discernible near the band edges, most clearly in BrSbS and BrSbSe and weaker in BrSbTe, indicating noticeable s–p mixing on the Sb site. Around the conduction-band minimum, Sb-p remains the leading contribution with additional weight from Sb-s and X s/p channels; the degree of this s–p hybridization diminishes toward Te, consistent with a progressive weakening of Sb–X covalency. Br-derived states contribute more selectively: Br-p and Br-d projections appear at specific **k**-segments but are comparatively small at the immediate band edges. This **k**-selective visibility is compatible with symmetry selection and structure-factor effects that permit Br channels to mix only for compatible irreducible representations and constructive Bloch phases; consequently, weight can be enhanced near energetic alignment with Sb/X-derived bands and suppressed at high-symmetry points where mixing is symmetry-forbidden. Co-occurrence of Br-p and Br-d weight at the same **k** and energy is therefore suggestive but not by itself conclusive of hybridization without evidence of avoided crossings or a smooth exchange of character along the dispersion. Across the series, the fundamental gap reduces from S to Se to Te, and the momentum offset between the valence and conduction edges becomes more pronounced in BrSbTe than in BrSbS/BrSbSe, which appear nearly direct along the plotted path. Overall, the projected bands indicate that Sb-p and X-p orbitals dominate the frontier states, with a non-negligible, composition-dependent Sb-s participation, while Br-p and Br-d contributions are present in a symmetry- and **k**-dependent manner but remain secondary near the gap edges.

### 3.3 Elastic properties and mechanical stability

The elastic behaviour of the material is essentially governed by its mechanical strength, flexibility and piezoelectricity. In two-dimensional (2D) materials, elastic constants directly affect the piezoelectricity response of the lattice strain *d*_11_ to *d*_11_ = *e*_11_/(*C*_11_ − *C*_12_), and also contribute to the phonon group velocities that are important for the thermal conductivity of the lattice. For two-dimensional materials with trigonal symmetry (*P*3*m*1 space group), the elastic response is therefore not only valid for the mechanical stability of the monolayers, but also a condition for the interpretation of the piezoelectricity and thermal transport properties. For a monolayer, only the in-plane strain components (*ε*_1_, *ε*_2_, *ε*_6_) and the out-of-plane components (*ε*_3_, *ε*_4_, *ε*_5_) are relevant. In this case, the in-plane tensor of the elasticity of the plane *C*_*ij*_ is reduced to a symmetric matrix of 3 × 3:7
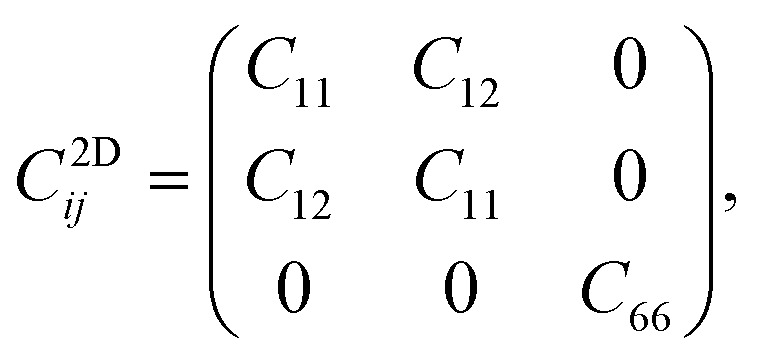
where 
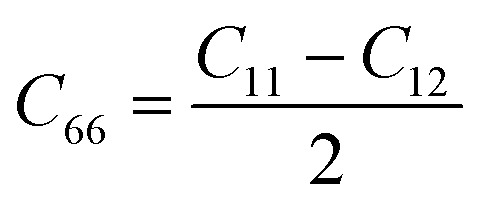
 in the case of trigonal systems. All three BrSbX monolayers are confirmed to be mechanically stable, as demonstrated by their elastic tensors, which meet the two-dimensional mechanical stability criteria of the Born–Huang test: *C*_11_ > 0 and *C*_11_ > |*C*_12_|.^57^ In addition, their positive eigenvalues in the matrix of stiffness further enhance dynamic stability in accordance with the results of phonon dispersion. Table 1 summarises the main elastic constants and derived properties. In particular, BrSbS has the lowest elastic modulus and the lowest shear modulus of all three, with *E*^2D^ = 30.014 N m^−1^ and *G* = 10.878 N m^−1^. Although mechanically softer, BrSbS shows a relatively higher Poisson's ratio of 0.38 compared to 0.31 and 0.26 for BrSbSe and BrSbTe, respectively. This enhanced compliance may play a role in facilitating larger piezoelectric responses because of its lower resistance to in-plane deformation. Although mechanically softer by Young's modulus (*E*^2D^ = 30.014 N m^−1^), BrSbS has a slightly larger *C*_11_ (35.06 N m^−1^) than BrSbTe (34.44 N m^−1^). BrSbTe shows the largest *E*^2D^ (32.095 N m^−1^). We therefore report both *E*^2D^ and (*C*_11_, *C*_12_) to avoid conflating these measures. The bulk-to-shear ratio *K*/*G* is systematically decreasing from BrSbS (2.223) to BrSbTe (1.706), suggesting a trend towards increased shear resistance and reduced ductility with heavier substitution of chalcogen. The calculated values of elastic constants for all three compounds are perfectly isotropic within the basal plane (anisotropy index = 0), consistent with their high-symmetry trigonal *P*3*m*1 space group.

**Table 1:** Elastic properties of BrSbX (X = S, Se, Te) monolayers. All values are in N m^−1^ except the Poisson's ratio (dimensionless)

Material	Young's modulus *E*	Shear modulus *G*	Poisson's ratio *ν*	Bulk modulus *K*	Bulk/Shear ratio *K*/*G*
1T-BrSbS	30.014	10.878	0.380	24.186	2.223
1T-BrSbSe	30.512	11.637	0.311	22.143	1.903
1T-BrSbTe	32.095	12.727	0.261	21.712	1.706

## 4 Piezoelectric properties of BrSbX

Piezo-electricity is central to electromechanical coupling phenomena and is the basis for the development of nanoscale sensors, actuators and energy-storage devices. In two-dimensional (2D) materials, the absence of inversion symmetry is a prerequisite for the existence of non-zero piezoelectric coefficients. All 1T-phase BrSbX monolayers (X = S, Se, Te) crystallize in the trigonal polar space group *P*3*m*1, which belongs to the non-centrosymmetric point group 3*m* and allows for the piezoelectric response in the plane. For 2D materials, mechanical deformations and the resulting polarization are usually confined to the basal plane (*xy*-plane), while the stress and strain out-of-plane components (*σ*_3_, *ε*_3_, *σ*_4_, *ε*_4_, *σ*_5_, *ε*_5_) are effectively nullified. Under this constraint, the linear piezoelectric effect is described by the reduced form of the third-rank piezoelectric stress tensor *e*_*ik*_, and its strain counterpart *d*_*ij*_, as follows for materials with trigonal symmetry 3*m*.8
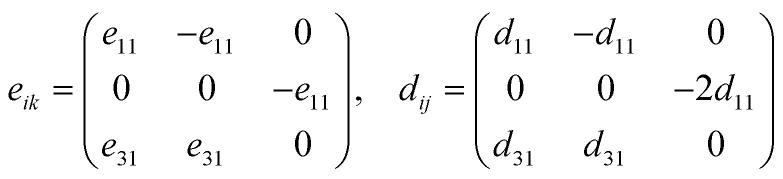
In this representation, only the components *e*_11_, *e*_31_, *d*_11_, and *d*_31_ are allowed symmetry. The out-of-plane term *d*_31_, although often small, remains nonzero in the absence of mirror symmetry with respect to the *z*-axis. The strain piezoelectric coefficients are related to their stress counterparts *via* the elastic stiffness constants. Specifically, for *d*_11_ and *d*_31_, the relations are given by:9
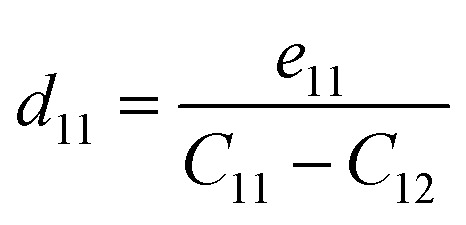
10
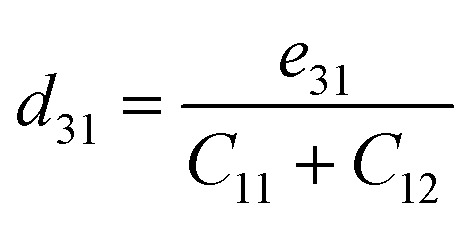


Here, *C*_11_ and *C*_12_ are the in-plane elastic constants of the 2D crystal, and *e*_11_, *e*_31_ are the piezoelectric stress tensor components. Piezoelectric coefficients obtained for the 1T-BrSbX compounds are summarized in Table 2. The piezoelectric stress constant *e*_11_ is systematically decreasing from 8.93 × 10^−10^ C m^−1^ for BrSbS, to 5.15 × 10^−10^ C m^−1^ in BrSbSe, and down to 1.60 × 10^−10^ C m^−1^ for BrSbTe. Using the elastic constants discussed above (*e.g.*, *C*^BrSbS^_11_ = 35.06 N m^−1^ and *C*^BrSbS^_12_ = 13.31 N m^−1^). The piezoelectric strain coefficients in the plane *d*_11_ were determined to be 6.27 pm V^−1^ for BrSbTe, 22.14 pm V^−1^ for BrSbSe, and 41.04 pm V^−1^ for BrSbS which in good agreement with literature.^58^ Both a weakening of the polarizable Sb–X bonds and a decrease in the polarization strength (observed in the charge redistribution and COHP plots) are associated with the decline in piezoelectric response throughout the chalcogen series. Under a consistent thickness convention, the 1T-BrSbX coefficients are competitive with leading 2D piezoelectrics reported in the literature, surpassing traditional systems like MoS_2_ (3.6 pm V^−1^) and group-III nitrides in monolayer form.^58^ It should be noted that the calculated 2H-BrSbX piezoelectricity is much higher, as shown in Table 2 and the previous study, but since it is noted that the 1T structure has a lower formation energy, which suggests that it is likely to form more easily in experiments, this work is focused on the 1T structure. Additional density functional perturbation theory calculations of the Born effective charges are provided in the SI and confirm the bonding-based interpretation of the piezoelectric trends discussed here.

**Table 2:** Comparison of structural, piezoelectric, and elastic properties of BrSbX (X = S, Se, Te) in 1T and 2H phases. Literature values^58^ are denoted with superscripts ^*a*^ for *a*, *e*_11_, and *d*_11_, and ^*b*^ for *C*_11_, *C*_12_

Phase	Compound	*a* (Å)	*e*_11_ (10^−10^ C m^−1^)	*C*_11_ (N m^−1^)	*C*_12_ (N m^−1^)	*d*_11_ (pm V^−1^)
1T	BrSbS	3.99 (3.99*^a^*)	8.93 (8.99*^a^*)	35.06 (33.76*^b^*)	13.31 (12.77*^b^*)	41.04 (42.84*^a^*)
1T	BrSbSe	4.08 (4.08*^a^*)	5.15 (5.10*^a^*)	33.78 (34.11*^b^*)	10.51 (8.44*^b^*)	22.14 (19.87*^a^*)
1T	BrSbTe	4.24 (4.24*^a^*)	1.60 (1.56*^a^*)	34.44 (31.29*^b^*)	8.99 (6.89*^b^*)	6.27 (6.41*^a^*)
2H	BrSbS	3.88 (3.88*^a^*)	11.88 (11.82*^a^*)	27.29 (27.08*^b^*)	25.13 (23.91*^b^*)	547.91 (373.00*^a^*)
2H	BrSbSe	3.97 (3.97*^a^*)	9.78 (9.82*^a^*)	27.59 (27.09*^b^*)	20.44 (19.57*^b^*)	136.82 (130.55*^a^*)
2H	BrSbTe	4.12 (4.11*^a^*)	8.22 (8.25*^a^*)	26.81 (26.49*^b^*)	15.35 (14.62*^b^*)	71.72 (69.54*^a^*)

### 4.1 Lattice dynamics and thermal properties

**Fig. 4:**
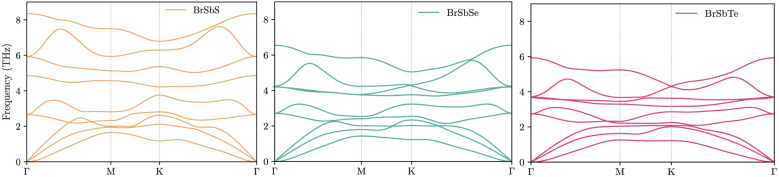
Phonon dispersion curves of 1T-phase BrSbX (X = S, Se, Te) monolayers computed using density functional perturbation theory. The phonon spectra are plotted along high-symmetry directions *Γ* → M → K → *Γ* in the 2D Brillouin zone. All compounds exhibit dynamical stability, as evidenced by the absence of imaginary frequencies. A systematic softening of phonon modes is observed from BrSbS to BrSbTe, associated with the increasing atomic mass of the chalcogen element.

Fig. 4 shows that all three monolayers are dynamically stable with no imaginary branches. The acoustic sector exhibits the expected quadratic flexural mode (ZA) near *Γ* and linearly dispersing in-plane modes (TA, LA). The slopes of the LA branch at *Γ* indicate a progressive reduction of sound velocity from BrSbS to BrSbTe, consistent with harmonic softening as the chalcogen mass increases (about 3.41, 3.12, and 2.78 km s^−1^ for S, Se, and Te, respectively). Concomitantly, the acoustic-optical separation narrows from S to Te and several avoided crossings emerge along M–K, signaling enhanced mixing between low-lying optical modes and acoustic branches in the heavier compound. These dispersion features align with the projected phonon density of states, where low-frequency weight grows toward Te and out-of-plane components dominate the acoustic window. Importantly, the mode-resolved Grüneisen parameters are largest for BrSbS in the long-wavelength acoustic sector, which shortens lifetimes and suppresses heat conduction despite the higher group velocities; BrSbTe displays reduced acoustic anharmonicity and longer lifetimes, explaining its larger lattice thermal conductivity. The calculated phonon dispersion using DFT and MLIP is shown in Fig. S1 of the SI. A slight deviation is observed between MLIP and DFT in the second-highest optical phonon branch near the *Γ* point, where MLIP slightly underestimates the frequency. This is attributed to the sensitivity of high-frequency optical modes to long-range force constants and underrepresentation in the training set. However, the discrepancy is minor and localized and does not affect the broader accuracy of the MLIP model.

**Fig. 5:**
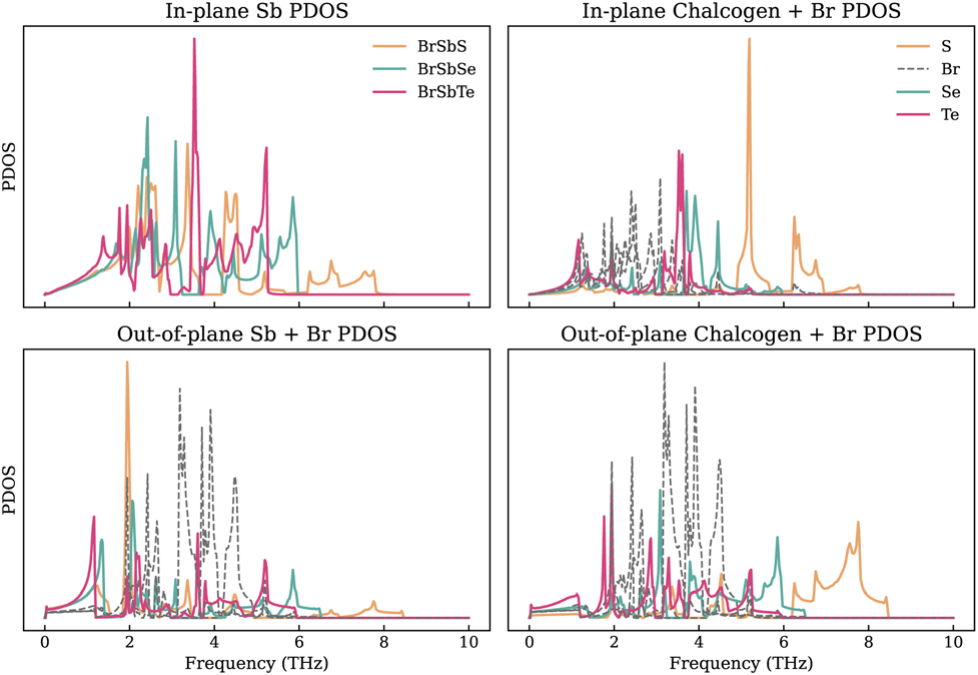
Projected phonon density of states (PDOS) for 1T-BrSbX (X = S, Se, Te) along the series. For each composition, the total PDOS is shown together with element-resolved contributions and in-plane/out-of-plane polarizations. All spectra are normalized such that the integral equals 3*N* per unit cell. From S to Te, the PDOS exhibits a systematic redshift and a narrowing of the acoustic–optical separation, consistent with the larger chalcogen mass and harmonic softening. Low-frequency weight increases toward Te, and enhanced overlap between Br and chalcogen contributions indicates stronger mixing of out-of-plane motions in the acoustic window.

Thermoelectric performance is quantified by 
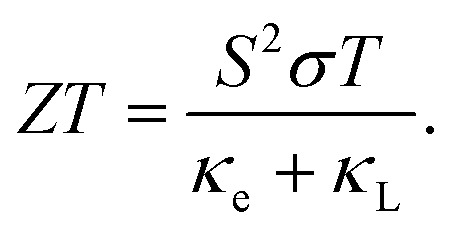
 The electronic thermal conductivity obeys the Wiedemann–Franz relation, *κ*_e_ ≈ *LσT*, which couples *κ*_e_ directly to *σ*. By contrast, the lattice contribution *κ*_L_ arises from phonon transport and, to leading order, is independent of *σ*. We therefore focus on *κ*_L_ in this analysis. Our analysis reveals a systematic increase of *κ*_L_ from S → Se → Te, governed by the competition between harmonic softening (lower *v*_g_) and anharmonic scattering (shorter *τ* toward S). First, phonon group velocities (*v*_g_) are significantly reduced with increasing chalcogen mass. As shown by phonon dispersion calculations, the maximum velocity of the primary heat-carrying longitudinal acoustic (LA) mode drops from approximately 3.41 km s^−1^ in BrSbS to below 2.78 km s^−1^ in BrSbTe, as shown in Fig. 7. This “softening” of the lattice is directly caused by the unit cell's increased average atomic mass, which slows the propagation of vibrational waves. Since *κ*_L_ is proportional to *v*^2^_g_, this effect alone provides a substantial reduction in thermal transport.

Second, and more critically, the Grüneisen parameters resolved in mode reveal that BrSbS exhibits the highest values *γ*, particularly in low-frequency acoustic modes. This indicates that BrSbS is the most anharmonic in the series, leading to strong phonon–phonon scattering and short phonon lifetimes (*τ*). In contrast, BrSbTe shows lower *γ* values, corresponding to weaker scattering and longer phonon lifetimes. As a result, despite its heavier mass and lower group velocities, BrSbTe maintains the highest *κ*. BrSbSe lies in between, with intermediate *γ* values and conductivity. This competition between harmonic softening and anharmonic scattering explains the observed trend *κ* across the series. In phonon-dominated 2D crystals, anharmonicity is commonly characterized, though not uniquely quantified, by the mode Grüneisen parameter^59^11
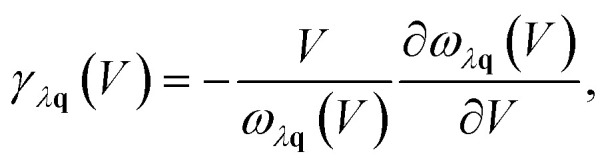
and condensed into a single descriptor by averaging over modes with their constant-volume heat-capacity weights:12
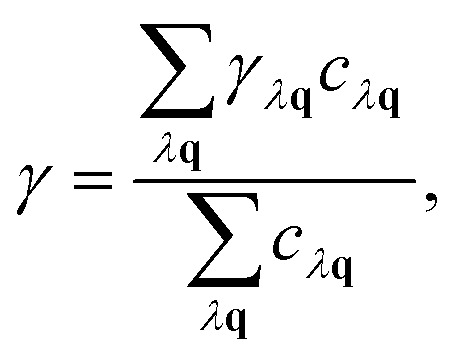
where *c*_*λ***q**_ is the modal heat capacity. The mean values for Janus BrSbX are *γ*(BrSbS) = 0.677, *γ*(BrSbSe) = 0.439, *γ*(BrSbTe) = 0.361. The resulting order *γ*_BrSbS_ > *γ*_BrSbSe_ > *γ*_BrSbTe_ indicates a monotonic decrease in effective anharmonicity from S to Te, consistent with the comparatively higher lattice thermal conductivity observed for BrSbTe.

**Fig. 6:**
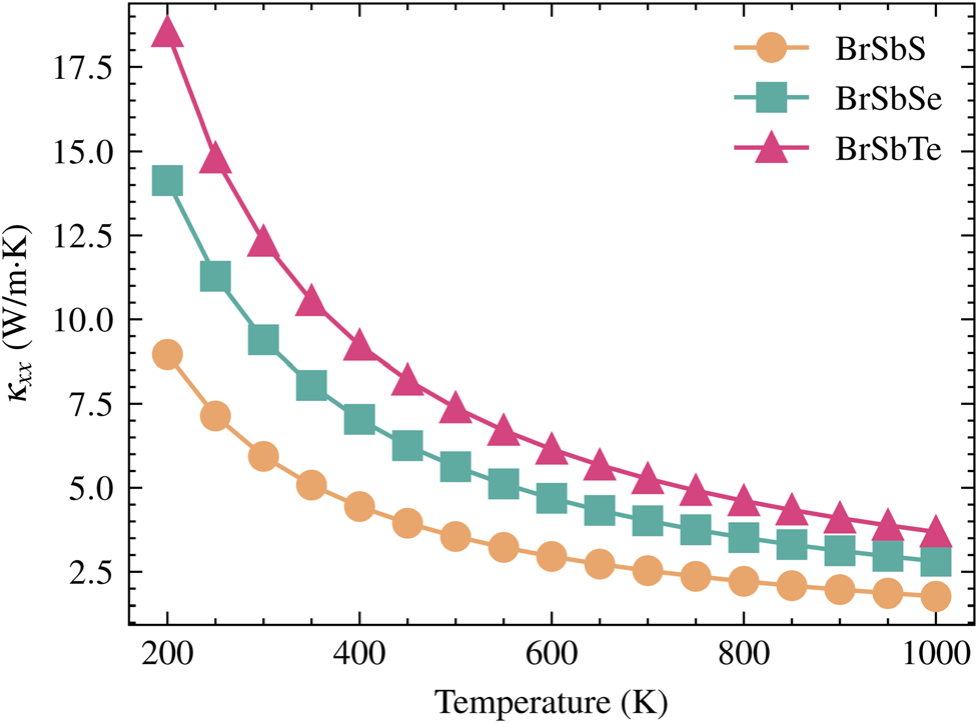
Temperature-dependent lattice thermal conductivity *κ*_*xx*_ of BrSbS, BrSbSe, and BrSbTe monolayers calculated using the Boltzmann transport equation within the relaxation time approximation. BrSbS exhibits the lowest *κ*_L_ values across all temperatures due to stronger anharmonic phonon scattering, while BrSbTe maintains the highest *κ*_L_ owing to its comparatively weaker lattice anharmonicity and reduced phonon scattering.

Analysis of the computational results for BrSbX monolayers reveals a clear trend: as the chalcogen atom changes from S to Se to Te, there is a systematic decrease in phonon group velocity and an decrease in lattice anharmonicity. The chalcogen's increasing atomic mass, which softens the phonon branches and lessens the lattice's stiffness, is directly responsible for this decrease. Mode-averaged Grüneisen parameters follow *γ*_BrSbS_ > *γ*_BrSbSe_ > *γ*_BrSbTe_, indicating decreasing effective anharmonicity from S to Te. In BrSbS, the larger mode-averaged Grüneisen parameters shorten acoustic phonon lifetimes; toward Te, weaker anharmonicity lengthens lifetimes. The combined effect of these trends is reflected in the calculated lattice thermal conductivities at 300 K: *κ*_L_ = 5.94 W m^−1^ K^−1^ for BrSbS, 9.39 W m^−1^ K^−1^ for BrSbSe, and 12.32 W m^−1^ K^−1^ for BrSbTe (Fig. 6). Solutions of the phonon Boltzmann transport equation within the relaxation-time approximation indicate that *κ*_L_ increases with the chalcogen mass (S → Se → Te). Although BrSbS exhibits the highest LA group velocities, its larger mode-averaged Grüneisen parameters signal stronger anharmonicity and hence shorter phonon lifetimes, which suppress *κ*_L_ and outweigh the velocity advantage. The cumulative *κ*_L_(*ω*) further shows that acoustic phonons dominate thermal transport, with optical modes contributing only marginally. It worths to note that, the lattice thermal conductivity of Janus monolayers is not universally lower than that of their symmetric parents. Our study focuses on how vertical chemical asymmetry within BrSbX controls bonding and anharmonicity, rather than on comparison with the centrosymmetric parent phases.

**Fig. 7:**
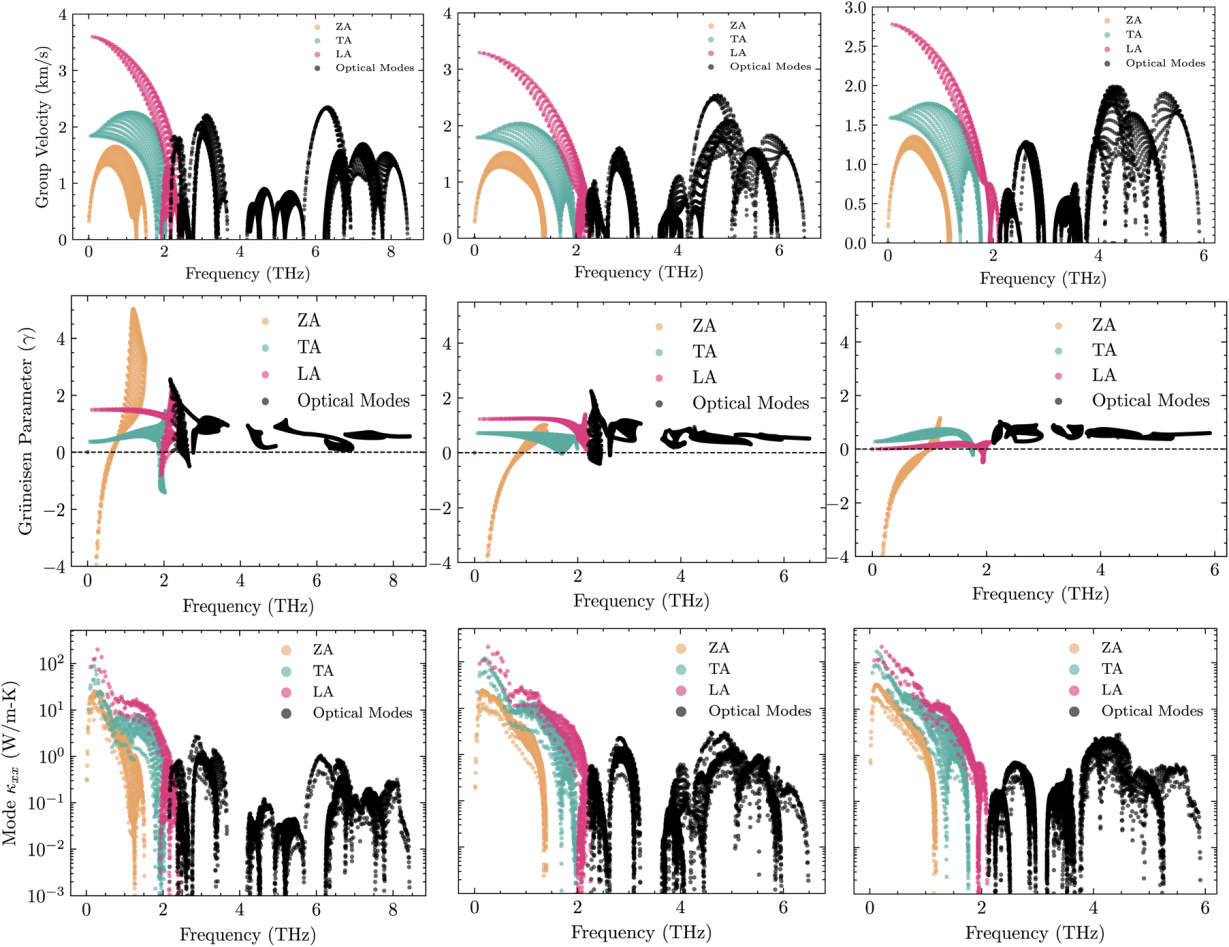
(a) Mode group velocities *v*_g_(**q**, *ν*) = *∂ω*_**q***ν*_/*∂***q** along *Γ*–M–K–*Γ* for BrSbX (X = S, Se, Te). The slopes of the longitudinal-acoustic (LA) branch near *Γ* decrease from S to Te (steeper for S, flatter for Te), reflecting harmonic softening with heavier chalcogens. (b) Mode-resolved Grüneisen parameters. Larger acoustic-branch *γ* in BrSbS and smaller values in BrSbTe indicate decreasing effective anharmonicity along S → Se → Te. (c) Modal lattice-thermal-conductivity contributions *κ*_**q***ν*_(*T*) obtained from the Boltzmann transport equation within the relaxation-time approximation at the reported temperature (see Methods). Acoustic modes dominate *κ* in all cases; reduced acoustic *γ* and longer lifetimes toward Te yield larger *κ* despite lower group velocities.

In order to comprehend the lattice dynamics and vibrational characteristics of BrSbX monolayers, we examine the projected phonon density of states (PDOS), shown in Fig. 5. A systematic redshift of vibrational frequencies is observed across the series from S to Te, reflecting phonon softening due to increasing atomic mass. Heavier atoms compress the dispersions and lower group velocities, with the maximum LA mode velocity dropping from ∼3.4 km s^−1^ in BrSbS to below 2.8 km s^−1^ in BrSbTe. This harmonic softening alone would suggest that the thermal conductivity should decrease from S to Te. However, the decisive factor is lattice anharmonicity. The mode-resolved Grüneisen parameters reveal that BrSbS exhibits the largest *γ* values, particularly in low-frequency ZA and TA modes. These strong anharmonic interactions drastically shorten the phonon lifetimes and suppress heat transport. The exceptionally low thermal conductivity of BrSbS also reflects strong acoustic–optical coupling (Fig. 4), which increases three-phonon scattering and reduces phonon lifetimes. This effect is significantly weaker in BrSbSe and BrSbTe. By contrast, BrSbTe shows lower *γ* values, indicating weaker phonon–phonon scattering. Although its phonon group velocities are lower, the reduced scattering allows phonons in BrSbTe to live longer and conduct heat more effectively. This competition between harmonic and anharmonic effects explains the observed ordering of thermal conductivities. BrSbS combines higher velocities with extremely strong anharmonic scattering, yielding the lowest *κ*. BrSbTe, despite softer dispersions, has weaker scattering and therefore sustains the highest *κ*. BrSbSe is intermediate, balancing both effects. The calculated room-temperature values are 5.94, 9.39, and 12.32 W m^−1^ K for BrSbS, BrSbSe, and BrSbTe, respectively, as shown in Fig. 6. Since only three-phonon processes are included, these *κ*_L_ values represent upper bounds; additional four-phonon scattering would further suppress *κ*_L_, especially at high temperature, but is not expected to alter the S → Se → Te trend governed by the interplay of group velocities and anharmonicity.

In the broader context of Janus materials, our findings reinforce and extend several trends. While Janus transition metal dichalcogenides (*e.g.*, MoSSe, WSSe) have shown moderate piezoelectric coefficients on the order of 2.26–5.3 pm V^−1^^60^ and high lattice thermal conductivities (342.5 W mK^−1^ for MoSSe,^61^ 33.6W mK^−1^ for ZrSSe,^62^ a few pm V^−1^ for Cr based janus^63^), our results reveal that BrSbS exhibits an exceptionally high *d*_11_ (41 pm V^−1^) together with much lower *κ* (<6 W mK^−1^). This combination of strong piezoelectric response and suppressed lattice thermal transport is rare among Janus compounds. Meanwhile, BrSbTe maintains relatively high *κ* values despite heavier mass, due to weaker anharmonicity, suggesting possible use in thermal management where efficient heat dissipation is desired. Taken together, these comparisons emphasize that BrSbX compounds enrich the Janus family by offering distinct trade-offs between piezoelectricity and thermal transport, and position them as versatile candidates across thermoelectric and piezoelectric device platforms. From a comparative perspective, BrSbX complements Janus TMDCs by revealing a regime where lattice thermal transport is governed predominantly by anharmonic scattering rather than harmonic softening. In BrSbS, large mode-resolved Grüneisen parameters in low-frequency acoustic modes shorten phonon lifetimes and suppress *κ*_*xx*_, whereas BrSbTe exhibits smaller *γ* and correspondingly longer lifetimes despite heavier mass-consistent with higher *κ*_*xx*_. These observations align with theoretical expectations for three-phonon Umklapp processes and mode-specific scattering phase space. Concurrently, the in-plane piezoelectric coefficient of BrSbS (on the order of ∼41 pm V^−1^) sits at the high end among 2D materials and compares favorably with Janus TMDC reports.^5,8,13,17,64,65^ Methodologically, the use of ML interatomic potentials together with Phono3py/HiPhive-style workflows enables accurate third-order force constants without imposing real-space cutoffs during the Boltzmann transport evaluation, which can otherwise bias scattering at low frequency; our approach follows community best practices.

## 5 Conclusion

In summary, we have systematically investigated the structural, electronic, elastic, piezoelectric, and lattice thermal transport properties of 2D BrSbX monolayers (X = S, Se, Te) using the first-principles density functional theory. All compounds crystallize in a 1T-type trigonal structure and satisfy mechanical and dynamic stability criteria. The central finding of our investigation is that BrSbS exhibits the lowest lattice thermal conductivity of the series, a result that is counter-intuitive when considering only atomic mass trends, which would otherwise predict the heaviest compound, BrSbTe, to be the poorest thermal conductor. Although a simple mass argument would suggest the heaviest member (BrSbTe) should have the lowest *κ*_L_, we obtain the opposite ordering *κ*_S_ < κ_Se_ < *κ*_Te_ due to stronger anharmonic scattering in BrSbS. BrSbS exhibits the largest mode-resolved Grüneisen parameters and strongest anharmonic scattering, giving the lowest *κ*. In addition, BrSbS also possesses the highest piezoelectric coefficient *d*_11_ = 41.04pm V^−1^, compared to 22.14 and 6.27 pm V^−1^ for BrSbSe and BrSbTe, respectively. These combined properties indicate that BrSbS not BrSbTe is the most compelling candidate for piezoelectric-coupled thermoelectric applications among the three. This study underscores the importance of a holistic evaluation that integrates elastic, anharmonic, and electronic transport parameters rather than relying solely on low thermal conductivity as a predictor of thermoelectric performance. Our results offer a more rigorous framework for screening and designing 2D multifunctional materials, in addition to updating our understanding of BrSbX materials.

## Conflicts of interest

The authors declare that they have no conflict of interest.

## Supplementary Material

RA-016-D5RA08461J-s001

## Data Availability

The data supporting this article have been included as part of the supplementary information (SI). Supplementary information is available. See DOI: https://doi.org/10.1039/d5ra08461j.
