# Supplementary material for: Competing bonding and anharmonicity control piezoelectricity and thermal transport in janus BrSbX monolayers
Source: RSC Adv. 2026 Jan 30;16(7):6366–76. doi: 10.1039/d5ra08461j (PMC12857538; doi:10.1039/d5ra08461j)
Supplement: RA-016-D5RA08461J-s001 [file RA-016-D5RA08461J-s001.pdf]

Supplementary Information (SI) for RSC Advances.
This journal is © The Royal Society of Chemistry 2026

Supplementary Information: Competing bonding and anharmonicity control piezoelectricity and thermal transport in Janus BrSbX monolayers

Viet-Ha Chu,[1] Nguyen Quang Hai,[1] Pham Mai An,[1] Luong Minh Tuan,[2] Truong-Tho Pham,[3] and Duc-Long Nguyen[4,5]

[1]*Department of Physics, TNU-University of Education, Thai Nguyen, 250000, Vietnam*

[2]*HaNoi University of Civil Engineering, Vietnam*

[3]*Laboratory of Magnetism and Magnetic Materials, Science and Technology Advanced Institute, Van Lang University, Ho Chi Minh City, Vietnam*

[4]*Simulation in Materials Science Research Group, Science and Technology Advanced Institute, Van Lang University, Ho Chi Minh City, Vietnam*

[5]*Faculty of Applied Technology, Van Lang School of Technology, Van Lang University, Ho Chi Minh City, Vietnam*

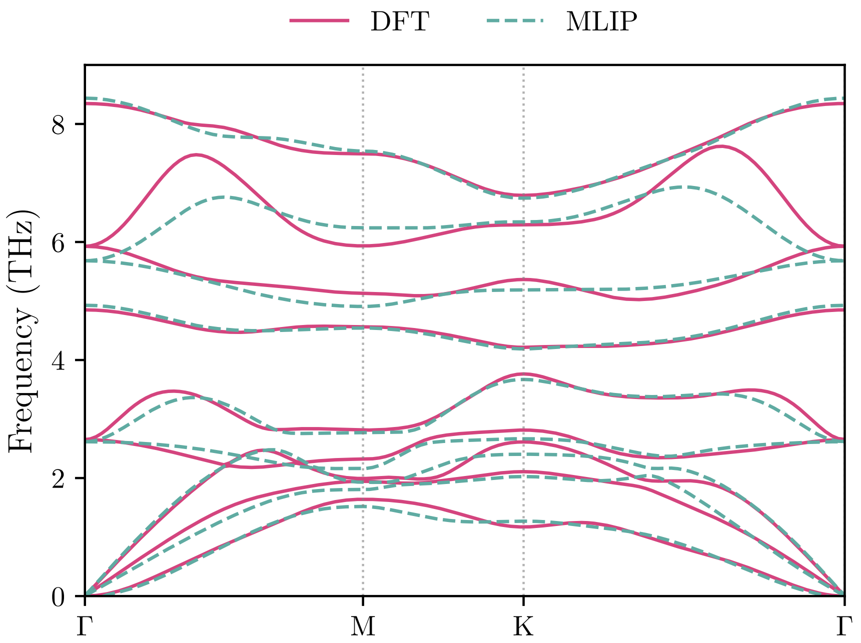

Fig. S 1. Phonon dispersion of BrSbS: DFT vs. MLIP. Phonon spectra along Γ–M–K–Γ computed using density-functional theory (DFT) and the trained machine-learning interatomic potential (MLIP). The MLIP reproduces the DFT acoustic and low-frequency optical branches accurately, and the largest deviation occurs in a localized high-frequency optical branch with a maximum difference of only a few cm^{-1}. These deviations are consistent with the MLIP validation statistics: energy MAE = 8.58 meV per configuration (0.114 meV/atom), RMS force error = 0.0268 eV $Å^{-1}$ (8.28% of the DFT RMS force), and maximal absolute force difference = 0.163 eV $Å^{-1}$. The validated MLIP is used to sample third-order force constants for the thermal-transport calculations.

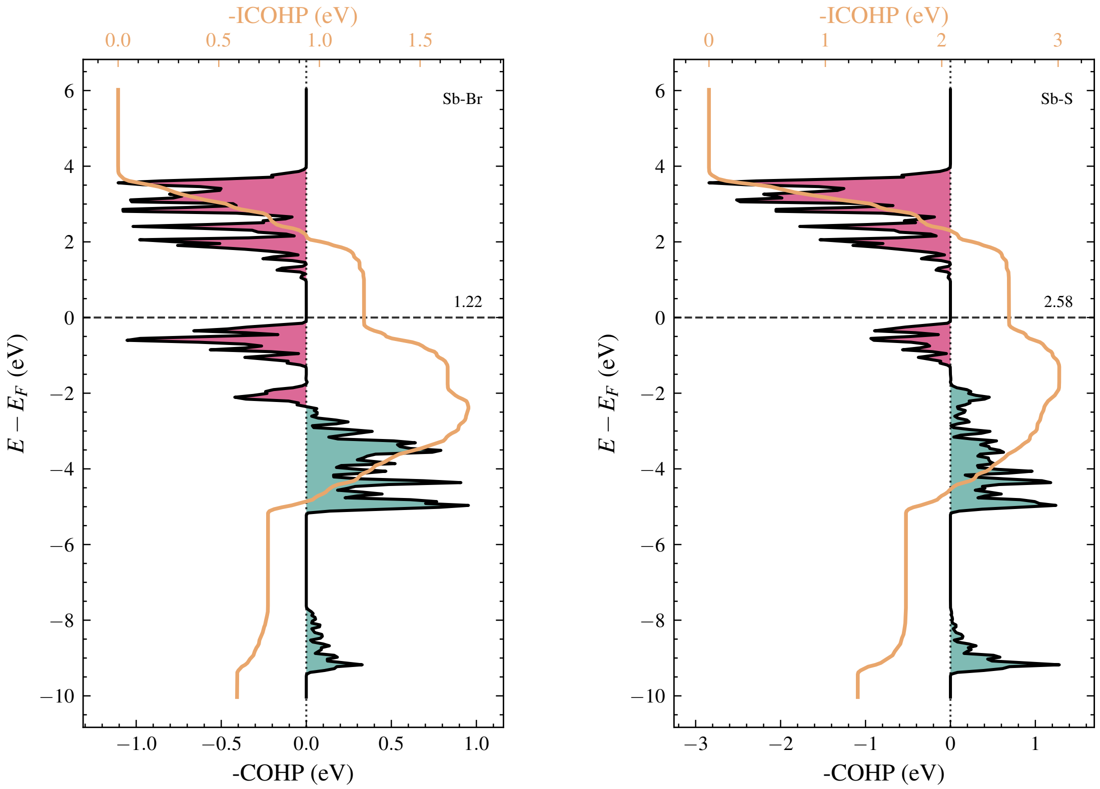

Fig. S 2. COHP and integrated −ICOHP for Sb–Br (left) and Sb–S (right) in BrSbS. Crystal orbital Hamilton population (COHP) referenced to the Fermi level ($E - E_F$), together with the integrated −ICOHP. Relative to Sb–S (Fig. S3), the Sb–Br bond shows weaker directional covalency, consistent with the bonding–property link discussed in the main text. Across the BrSbX series (X = S, Se, Te), the overall evolution is consistent with a progressive weakening of directional covalency from S to Te that moderates the polarization response under in-plane strain.

BORN EFFECTIVE CHARGES

To provide microscopic insight into the ionic origin of the piezoelectric response discussed in the main text, we computed the Born effective charge tensors of BrSbX

(X = S, Se, Te) monolayers using density functional perturbation theory. The diagonal in-plane components of the Sb Born effective charge decrease monotonically from BrSbS to BrSbSe and BrSbTe. In parallel, the magnitude of the Br in-plane Born effective charge decreases significantly, while that of the chalcogen X becomes more negative along the S-to-Se-to-Te sequence. These trends indicate a progressive reduction in dynamic charge transfer across the Sb–X bond, fully consistent with the COHP-based weakening of Sb–X covalency discussed in the main text. Importantly, this monotonic reduction of the effective charges directly correlates with the suppression of the in-plane piezoelectric coefficient reported in Table 2 of the main text. The DFPT Born effective charge results provide an independent, microscopic validation that the weakening of Sb-X covalency and internal-strain coupling governs the observed decrease of piezoelectric response from S to Se to Te.

TABLE I. Diagonal in-plane Born effective charges of BrSbX (X = S, Se, Te) monolayers obtained from DFPT. All values are given in units of the elementary charge.

System	Sb	Br	X
BrSbS	5.8896	−3.5868	−2.3028
BrSbSe	5.7149	−3.1060	−2.6089
BrSbTe	5.3395	−2.2047	−3.1348
